# Changes in ocular aquaporin expression following optic nerve crush

**Published:** 2010-03-03

**Authors:** Adnan Dibas, Hidehiro Oku, Masayuki Fukuhara, Takuji Kurimoto, Tsunehiko Ikeda, Rajkumar V. Patil, Najam A. Sharif, Thomas Yorio

**Affiliations:** 1 Department of Pharmacology & Neuroscience, University of North Texas Health Science Center at Fort Worth, Fort Worth, TX; 2 Department of Ophthalmology, Osaka Medical College, Osaka, Japan; 3 Department of Ophthalmology, Hyogo College of Medicine, Hyogo, Japan; 4 Pharmaceutical Research, Alcon Research Ltd, Fort Worth, TX

## Abstract

**Purpose:**

Changes in the expression of water channels (aquaporins; AQP) have been reported in several diseases. However, such changes and mechanisms remain to be evaluated for retinal injury after optic nerve crush (ONC). This study was designed to analyze changes in the expression of AQP4 (water selective channel) and AQP9 (water and lactate channel) following ONC in the rat.

**Methods:**

Rat retinal ganglion cells (RGCs) were retrogradely labeled by applying FluoroGold onto the left superior colliculus 1 week before ONC. Retinal injuries were induced by ONC unilaterally. Real-time PCR was used to measure changes in *AQP4*, *AQP9*, *thy-1*, *Kir4.1* (K^+^ channel), and β-actin messages. Changes in AQP4, AQP9, Kir4.1, B cell lymphoma-x (bcl-xl), and glial fibrillary acidic protein (GFAP) expression were measured in total retinal extracts using western blotting.

**Results:**

The number of RGCs labeled retrogradely from the superior colliculus was 2,090±85 cells/mm^2^ in rats without any treatment, which decreased to 1,091±78 (47% loss) and 497±87 cells/mm^2^ (76% loss) on days 7 and 14, respectively. AQP4, Kir4.1, and thy-1 protein levels decreased at days 2, 7, and 14, which paralleled a similar reduction in mRNA levels, with the exception of *Kir4.1* mRNA at day 2 showing an apparent upregulation. In contrast, *AQP9* mRNA and protein levels showed opposite changes to those observed for the latter targets. Whereas *AQP9* mRNA increased at days 2 and 14, protein levels decreased at both time points. *AQP9* mRNA decreased at day 7, while protein levels increased. *GFAP* (a marker of astrogliosis) remained upregulated at days 2, 7, and 14, while *bcl-xl* (anti-apoptotic) decreased.

**Conclusions:**

The reduced expression of *AQP4* and *Kir4.1* suggests dysfunctional ion coupling in retina following ONC and likely impaired retinal function. The sustained increase in GFAP indicates astrogliosis, while the decreased bcl-xl protein level suggests a commitment to cellular death, as clearly shown by the reduction in the RGC population and decreased *thy-1* expression. Changes in *AQP9* expression suggest a contribution of the channel to retinal ganglion cell death and response of distinct amacrine cells known to express *AQP9* following traumatic injuries.

## Introduction

The glaucomas represent a heterogeneous group of diseases that result in a progressive optic neuropathy characterized by functional and structural impairment of ocular tissues. Particularly affected are the trabecular meshwork, the optic nerve head, and the retinal ganglion cells.

One of the risk factors in primary open angle glaucoma (POAG) is an associated elevation in intraocular pressure (IOP) [1]. Elevation of IOP results in the blockade of axonal transport in animals as well as displacement of the optic nerve head [2-7]. Abnormalities in water balance play an important role in the pathophysiology of a variety of neurologic disorders. Neuronal activity is associated with a redistribution of water—shrinkage of the extracellular space around active synapses while enhancement of the extracellular space volume at more distant sites [8]. The discovery of aquaporins (AQPs) has provided a molecular basis for understanding water transport in several tissues, including the ocular system [9]. Using Reverse-transcription-Polymerase Chain Reaction (RT–PCR), Tenckhoff et al. showed that human retina expresses mRNAs for *AQP0* to *AQP12*, whereas rat retina has the mRNAs for *AQP0*, *AQP1*, *AQP3*, *AQP4*, *AQP5*, *AQP6*, *AQP7*, *AQP8*, *AQP9*, and *AQP11*. The mRNAs for *AQP2*, *AQP10*, and *AQP12* were not detected in the rat neural retina [10]. The same study reported that the human Müller cell line MIO-M1 [11] expressed mRNA for *AQP0*, *AQP1*, *AQP3*, *AQP4*, *AQP5*, *AQP7*, *AQP8*, *AQP9*, *AQP10*, and *AQP11* but not *AQP2*, *AQP6*, and *AQP12* [10]. *AQP0* is expressed in lens fiber [12] and rodent bipolar cells [13,14] and has been reported in distinct amacrines and ganglion cells of the rat retina [14]. While *AQP1* is expressed by cornea endothelium, ciliary and lens epithelia, trabecular meshwork [15], pigment epithelial cells [16], rodent amacrine cells [17,18], and photoreceptor cells [19], *AQP3* is detected in conjunctiva [20]. *AQP4* is expressed by ciliary epithelium and Müller cells and astrocytes [21-23], and *AQP5* has been reported in corneal and lacrimal gland epithelia [24]. *AQP9* has been detected in tyrosine hydroxylase-expressing amacrine cells in the rat retina [25], rat retinal ganglion cells (RGCs) [26], and human and rat pigment epithelial retinal cells [27].

Hypoxia and ischemia are associated with changes in the densities of AQP expression and are also considered key risk factors in the development of glaucomatous optic nerve neuropathy [1]. Interestingly, other insults, such as hypo-osmotic stress, known to affect AQP levels have also been reported to cause glaucoma [28-30]. However, changes in the expression of ocular AQPs upon retinal injuries have not been fully characterized. In the current study we analyzed changes in *AQP4* and *AQP9* following optic nerve crush (ONC). The crush model closely mimics the damage that occurs in traumatic optic neuropathy, which results from indirect trauma to the optic nerve. Such injuries appear to involve both mechanical (primary) and ischemic-induced (secondary) processes that include degeneration of nerve axons and loss of myelin. Many studies have observed loss of retinal ganglion cells following crush that is comparable to glaucoma, with the crush model regarded as an acute model of glaucoma [31-37].

## Methods

### Materials

Monoclonal anti-thy-1 and rabbit anti-kir4.1 antibodies were from Chemicon (Temecula, CA); AQP4 (mouse and rabbit), AQP9 (goat and rabbit), B cell lymphoma-x (bcl-xl; mouse), and thy-1 (rabbit) antibodies were from Santa Cruz Inc. (Santa Cruz, CA); monoclonal anti-glial fibrillary acidic protein antibodies were from Neomarkers (Fremont, CA); monoclonal anti-tubulin antibodies were from Upstate Inc. (Lake Placid, NY); and secondary antibodies (donkey anti-mouse-conjugated Alexa 633, goat anti-rabbit-conjugated Alexa 633, and goat anti-mouse-conjugated Alexa 488) were from Invitrogen Inc. (Carlsbad, CA).

### Animals

Nine-week-old male Wistar rats were purchased from Japan SLC (Shizuoka, Japan). The rats were housed in an air-conditioned room with a temperature of approximately 23 °C and 60% humidity and on a 12 h:12 h light–dark cycle. All animals were handled in accordance with the ARVO resolution for the Use of Animals in Ophthalmic and Vision Research. The experimental protocol was approved by the Committee of Animal Use and Care of the Osaka Medical College.

### Optic nerve crush

Eight animals were anesthetized with 7% chloral hydrate solution (400 mg/kg). After making a skin incision along the superior orbital margin, the superior surface of the right eye was exposed. Removing the superior rectus muscle exposed the optic nerve, which was crushed with forceps 2 mm behind the eye for 10 s, as described [38]. Care was taken not to cause retinal ischemia, and we confirmed by indirect ophthalmoscopy that the retinal circulation was not blocked. This procedure induced nearly complete damage to the optic nerve, and none of the RGCs could be retrogradely labeled from the superior colliculus [39]. A sham operation was performed on the right eyes of other animals, and the optic nerve was exposed in the same way but not crushed as in the experimental animals. The left eyes were not used as controls because crushing one optic nerve affects the morphology of the contralateral retina [40].

### Rat retinal ganglion cells labeling and counting

To determine how the RGCs were lost following ONC, RGCs were retrogradely labeled by applying FluoroGold (hydroxystilbamidine; Biotium, Hayward, CA) onto the left superior colliculus. Because no RGCs were labeled when the tracer was applied after the optic nerve was crushed, the tracer was applied 1 week before crushing the right optic nerve. After anesthesia with 7% chloral hydrate, a skin incision was made, the skull was exposed, and the bone over the left hemisphere was drilled along the sagittal, coronal, and lambdoidal sutures and removed. After the dura was peeled off, the gray and white matter of the left hemisphere was carefully removed by aspiration through the window, and the surface and brachium of the superior colliculus were exposed. Then, a small sponge soaked with 2% FluoroGold solution in saline containing 10% DMSO (DMSO) was placed directly onto the superior colliculus, as described [41]. Seven days after FluoroGold application, the right optic nerve was crushed. The operated rats were perfused transcardially with 4% paraformaldehyde in phosphate-buffered saline (PBS; 150 mM NaCl, 3.8 mM NaH_2_PO_4_, 16.2 mM Na_2_HPO_4_) on day 7 (n=4) and 14 (n=4) after the optic nerve crush, and the eyes were enucleated. The retinas were dissected, flatmounted, and examined under a fluorescence microscope (E800; Nikon, Tokyo, Japan) with a UV filter (365 nm).We counted the number of FluoroGold-labeled RGCs that had round-shaped somata and had fine regular dot-like fluorescent particles of FluoroGold in the cytoplasm. The number of RGCs was counted in 12 areas (0.48×0.48 mm^2^) at a distance of 1, 2, and 3 mm from the optic disc along the nasotemporal and dorsoventral midlines (upper, lower, nasal, and temporal direction). The mean density of RGC/mm^2^ was calculated, and the loss of RGCs was evaluated by comparing the density in the retinas with ONC to that in untreated retinas (n=4).

### Real-time analyses of *AQP4*/*AQP9*/*Kir4.1*/*thy-1*/*ACTB* mRNA levels

On days 2, 7, and 14, total RNA was isolated from the retina after the ONC or sham operation with Trizol (Life Technology, Grand Island, NY) as described by manufacturer’s instructions. Retina was removed and lysed directly by adding 1 ml Trizol and pipetting up and down several times. Two hundred μl of chloroform were added followed by inverting tubes for several times then samples were put on ice for 5 min. Tubes were then centrifuged for 10 min at 16,000× g and the upper 600 μl supernatant was removed to new tubes. Six hundred microliters of isopropanol were added followed by inversion of samples several times then samples were put on ice for 10 min. RNA was recovered by repeating centrifugation for 10 min at the same speed. Supernatant was discarded and RNA pellet was washed with 1 ml of 70% ethanol and centrifugation was repeated (10 min at 16,000× g). The ethanol solution was removed and RNA pellet was allowed to dry for 10 min at room temperature. In each tube, 50 μl TE buffer was added (10 mM Tris-HCl, pH 8, 1 mM EDTA) following by pipetting up and down several times to resuspend RNA. RNA concentration was determined by adding 6 μl from each sample in 600 μl water and measuring the absorbance at 260 nm (when absorbance is 1, the RNA concentration is 40 ng/ml). In this study, 2 µg of total RNA was reverse transcribed using the One-step reverse transcriptase (RT)–PCR kit (Qiagen, Valencia, CA), according to the manufacturers instructions. Control quantative (Q)-PCR reactions were performed in the absence of cDNA templates. β-actin (*ACTB*) was used as a housekeeping gene. The primers for retinal *AQP4* were 5′-CGG TTC ATG GAA ACC TCA CT-3′ (sense) and 5′-CAT GCT GGC TCC GGT ATA AT-3′ (antisense), giving a 191-bp amplicon. The primers for retinal *Kir4.1* were 5′-GCA AGA TCT CCC CCT CCG CAG-3′ (sense) and 5′-CAG ACG TTA CTA ATG CGC ACA CT-3′ (antisense), giving a 345-bp amplicon. The primers for *ACTB* were 5′-TGT GAT GGT GGG AAT GGG TCA G-3′ (sense) and 5′-TTT GAT GTC ACG CAC GAT TTC C-3′ (antisense), giving a 514-bp amplicon. The primers for retinal *AQP9* were 5′-CTC AGT CCC AGG CTC TTC AC-3′ (sense) and 5′-ATG GCT CTG CCT TCA TGT CT-3′ (antisense), giving a 184-bp amplicon. The primers for retinal *thy-1* were 5′-CGC TTT ATC AAG GTC CTT ACT C-3′ (sense) and 5′-GCG TTT TGA GAT ATT TGA AGG T-3′ (antisense), giving a 344-bp amplicon. A 2.5-µl sample of cDNA from each treatment was used for RT–PCR amplification of each primer in a Cepheid smart cycler (Cepheid, Sunnyvale, CA). For retinal *AQP4* and *Kir4.1*, it was 45 cycles of denaturation at 95 °C for 30 s, annealing at 60 °C for 30 s, and extension at 72 °C for 1 min. For retinal *AQP9*, it was 45 cycles of denaturation at 95 °C for 30 s, annealing at 60 °C for 30 s, and extension at 72 °C for 1 min. For retinal *thy-1*, it was 50 cycles of denaturation at 95 °C for 15 s, annealing at 55 °C for 10 s, and extension at 72 °C for 20 s. For *ACTB*, it was 40 cycles of denaturation at 95 °C for 1 min, annealing at 60 °C for 1 min, and extension at 72 °C for 2 min. The melting curves were generated to detect the melting temperatures of the specific products immediately after the PCR run. The relative mRNA levels were determined by the comparative cycle number at threshold (CT) method, as described in PE Biosystems User Bulletin #2. The fold change was determined by the formula: fold change=2–Δ(ΔC_t_), where ΔC_t_=C_t_,*target*-C_t_,β-actin, where “target” is the gene of interest. To verify the sequence of products, regular PCR was performed, and the PCR products were run on a 1.5% agarose gel in parallel with 100-bp DNA markers and stained with ethidium bromide. Bands were cut and sequenced to verify identity. The authenticity of PCR products was confirmed using a BLAST search of the sequence through the NCBI.

### Western blotting

Retinas from eight rats that underwent ONC and eight rats that underwent a sham operation were dissected. Western blotting was performed on either total retinal lysates or enriched plasma membrane and cytosolic fractions, as described by Dibas et al. 2008 [42]. To prepare total retinal lysate, retinas were dissected from eyes and solubilized in 250 µl of a solution containing 20 mM Tris (pH 7.4), 10% sucrose, 2 mM Ethylenediamine-tetraacetic acid (EDTA), 2 mM Ethylene glycol-bis(2-aminoethylether)-N,N,N′,N′-tetraacetic acid (EGTA), 50 mM NaF, 1% Triton X-100, 0.1% sodium dodecyl sulfate, and protease inhibitors (aprotonin (10 μg/ml), soybean trypsin inhibitor (10 μg/ml), leupeptin (10 μg/ml), and phenylmethanesulfonyl fluoride (100 μM)) at 4 °C. Retinal lysates were incubated for 30 min on ice, briefly sonicated before centrifugation at 14,000× g for 15 min, and the supernatant collected. Protein concentration was measured using a bicinchoninic acid (BCA) protein assay kit (Sigma, St. Louis, MO) with bovine serum albumin (BSA) as the standard. To enrich plasma membrane fractions, retinas were dissected from eyes and homogenized into 700 µl of a solution containing 20 mM Tris (pH 7.4), 10% sucrose, 2 mM EDTA, 2 mM EGTA, 50 mM NaF, and protease inhibitors at 4 °C, using a Potter homogenizer (Fisher Scientific, Houston TX, 60 strokes) followed by centrifugation at 3,000× g for 5 min. The unbroken tissue was sonicated 7X, and centrifugation was repeated. The retinal supernatant was then centrifuged for 30 min at 100,000× g at 4 °C. The resultant supernatant (enriched cytosol) and pellet (enriched plasma membrane) were collected for protein measurement using the BCA protein assay. Proteins were separated by 10% sodium dodecyl sulfate- PAGE (PAGE), with 20–30 µg (enriched fraction) or 100 µg (total retinal extract) of protein loaded in each lane. Gels were equilibrated in transfer buffer (192 mM glycine, 20% methanol, and 25 mM Tris-HCl, pH 8.3) for 10 min at room temperature and electroblotted on nitrocellulose membranes for 75 min at 100 V using a Bio-Rad (Hercules, CA) electroblotting unit. The membranes were dried at room temperature. Western blotting was performed using a chemiluminescent kit (Amersham Biosciences, Bedford, MA). Membranes were incubated with primary antibodies for 60 min (1 µg/ml) and with the horseradish peroxidase-conjugated secondary antibody for 30 min (1:10,000). Membranes were exposed to an X-ray film for 30 s, then 1 min then 5 min. X-ray film was removed at the end of each time point and immediately developed. Membranes were stripped and probed with anti-β-tubulin antibodies for normalization. Experiments were repeated three times. Band densities were quantified with image analysis software (Scion, Frederick, MD), and the intensity of AQP4/AQP9/thy-1/ glial fibrillary acidic protein (GFAP)/bcl-xl/kir4.1 bands was normalized for every sample relative to the intensity of the respective tubulin bands.

### Statistical analyses

Data are reported as means±standard error of the mean. All experiments were repeated at least three times with to three to four replicates per condition each time. Statistical significance was determined by using one-way ANOVA and Tukey multiple comparison tests at p<0.05.

## Results

### Reduction of rat retinal ganglion cells after optic nerve crush and correlation with *thy-1* mRNA and protein levels

**Figure 1:**
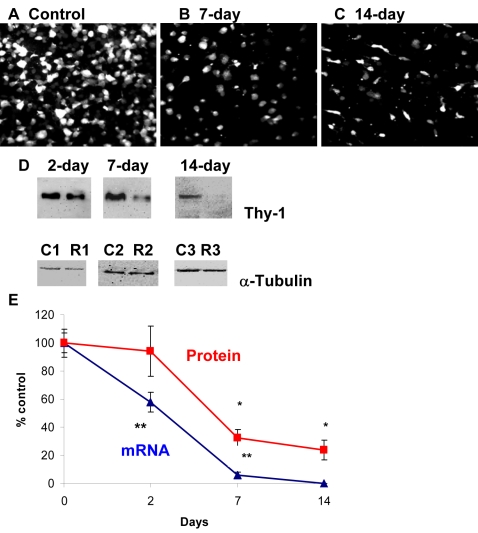
Optic nerve crush decreased number of retinal ganglion cells (RGCs), thy-1 protein, and mRNA levels. The mean number of RGCs labeled retrogradely from superior colliculus was 2090±85/mm^2^ in sham rats without any treatment (**A**), which decreased to 1091±78 (47% loss, **B**) and 497±87/mm^2^ (76% loss, **C**) on day 7 and 14, respectively. Thy-1 expressed by retinal ganglion cells is widely used as marker for RGC stress and was shown to be reduced following optic nerve crush. Following retinal injuries with optic nerve crush, retinas were dissected and plasma membrane proteins were isolated or total RNA was isolated and transcribed into cDNA. Thirty microgram protein was loaded into each lane. **D**: Immunoreactive bands for thy-1 and β-tubulin at 2, 7, and 14 days following crush showing a significant reduction in thy-1 protein levels at 7 and 14 day but not at 2 days (94±18%, 33±6%, 24±7% at 2, 7, and 14 day, respectively, n=7). Densitometric quantification is shown in **E**. Data are expressed as a ratio of the control value and each measurement represents mean±SEM *Denotes statistical significance of thy-1 protein levels in optic nerve crushed retinas versus sham (p<0.005) as determined by one-way ANOVA and Tukey multiple comparison test. Optic nerve crush also decreased thy-1 transcripts significantly (58±7%, 6±2%, at 2 and 7 day, respectively, % p<0.001 versus sham, n=8), as determined by quantitative real-time PCR (**E**). Thy1- mRNA at 2-weeks was below detection levels and considered zero. Gene expression data of thy-1 is calculated after normalizing with β-actin. **Denote significant differences compared with sham-retinas at p<0.05. Abbreviations: sham eye (C), and crushed (R).

ONC caused a massive reduction in the RGC population with time. While the mean number of sham RGCs was 2,090±85 cells/mm^2^ in rats (n=4, Figure 1A), the number decreased to 1,091±78 (n=4, 47% loss, Figure 1B) and 497±87 cells/mm^2^ (n=4, 76% loss versus sham, p<0.0001, Figure 1C) on days 7 and 14, respectively. Thy-1, a known RGC stress marker commonly used to measure RGC populations, was quantified. There was a reduction in thy-1 protein (94±18%, 33±6%, and 24±7% at days 2, 7, and 14, respectively, Figure 1D, p<0.005, n=7), although changes in thy-1 protein levels at day 2 were not statistically significant. In addition, ONC decreased *thy-1 *mRNA significantly (58±7% and 6±2%, days 2 and 7, respectively, p<0.001 versus sham, Figure 1E; data expressed as a ratio of the control value, n=8).* Thy-1 *mRNA at 2 weeks was below detection levels. Figure 1E shows parallel changes in *thy-1* mRNA to protein levels. When changes in *thy-1* expression were compared to the time course of RGC cell loss, it is clear that a significant loss of *thy-1* mRNA and protein occurs well in advance of RGC cell loss. For example, while the RGC population decreased by approximately 47% at day 7, *thy-1* mRNA decreased by 94%, showing that the reduction of *thy-1* mRNA is much greater than the loss of RGCs.

### Effect of optic nerve crush on *AQP4* in rat retinas

**Figure 2:**
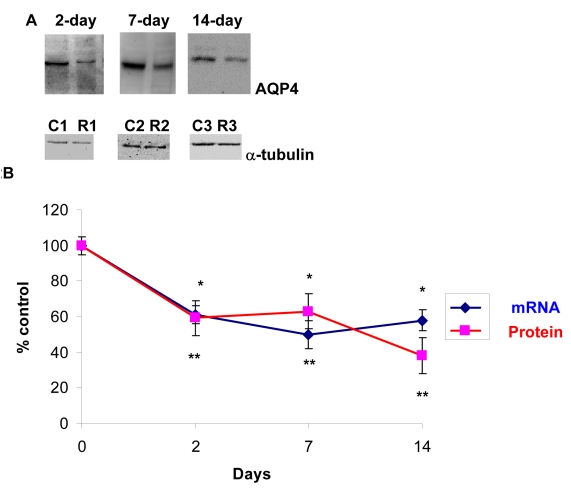
Optic nerve crush decreased AQP4 protein and mRNA levels. Following retinal injuries with optic nerve crush, retinas were dissected and plasma membrane proteins were isolated or total RNA was isolated and transcribed into cDNA. Thirty microgram protein was loaded into each lane. Immunoreactive bands for AQP4 and β-tubulin 2, 7, and 14 days after optic nerve crush showing a significant reduction in AQP4 protein levels. Quantitative measurement using western blot showed that optic nerve crush decreased AQP4 protein levels by ~40% (60±10, 63±10, 38±10, at 2, 7, and 14 day, respectively, n=7, **A**). Densitometric quantification is shown in **B**. Data are expressed as a ratio of the treated to control or sham value and each measurement represents mean±SEM *Denote statistical significance of AQP4 protein levels in optic nerve crushed retinas versus sham (p<0.005) as determined by one-way ANOVA and Tukey multiple comparison test. Optic nerve crush also decreased AQP4 transcripts significantly (61±5%, 60±8%, 58±6, at 2, 7, and 14 day, respectively, n=7), as determined by quantitative real-time PCR (**B**). Gene expression data of *AQP4* is calculated after normalizing with *ACTB*. **Denote significant differences compared with sham-retinas at p<0.05. Abbreviations: sham eye (C), and crushed (R).

Hypertrophy of the optic nerve has been reported in glaucoma subjects, and patients with normal tension glaucoma [43] or POAG [44] have a larger optic disc compared to normal patients. Therefore, the effect of ONC on *AQP4* expression in retina was tested. As shown in Figure 2A, ONC resulted in a decrease in AQP4 protein (60±10, 63±10, and 38±10, p<0.001 at days 2, 7, and 14, respectively, Figure 2A,B, n=7), and there as a similar reduction in* AQP4 *mRNA (~40%), as determined by Q-PCR (61±5%, 60±8%, and 58±6 at days 2, 7, and 14, respectively, p<0.001 versus control, Figure 2B, n=7). Figure 2B shows parallel changes in *AQP4* mRNA to protein levels. Data are expressed as a ratio of the control value, and each measurement represents mean±SEM.

### Effect of optic nerve crush on *Kir4.1* in rat retinas

**Figure 3:**
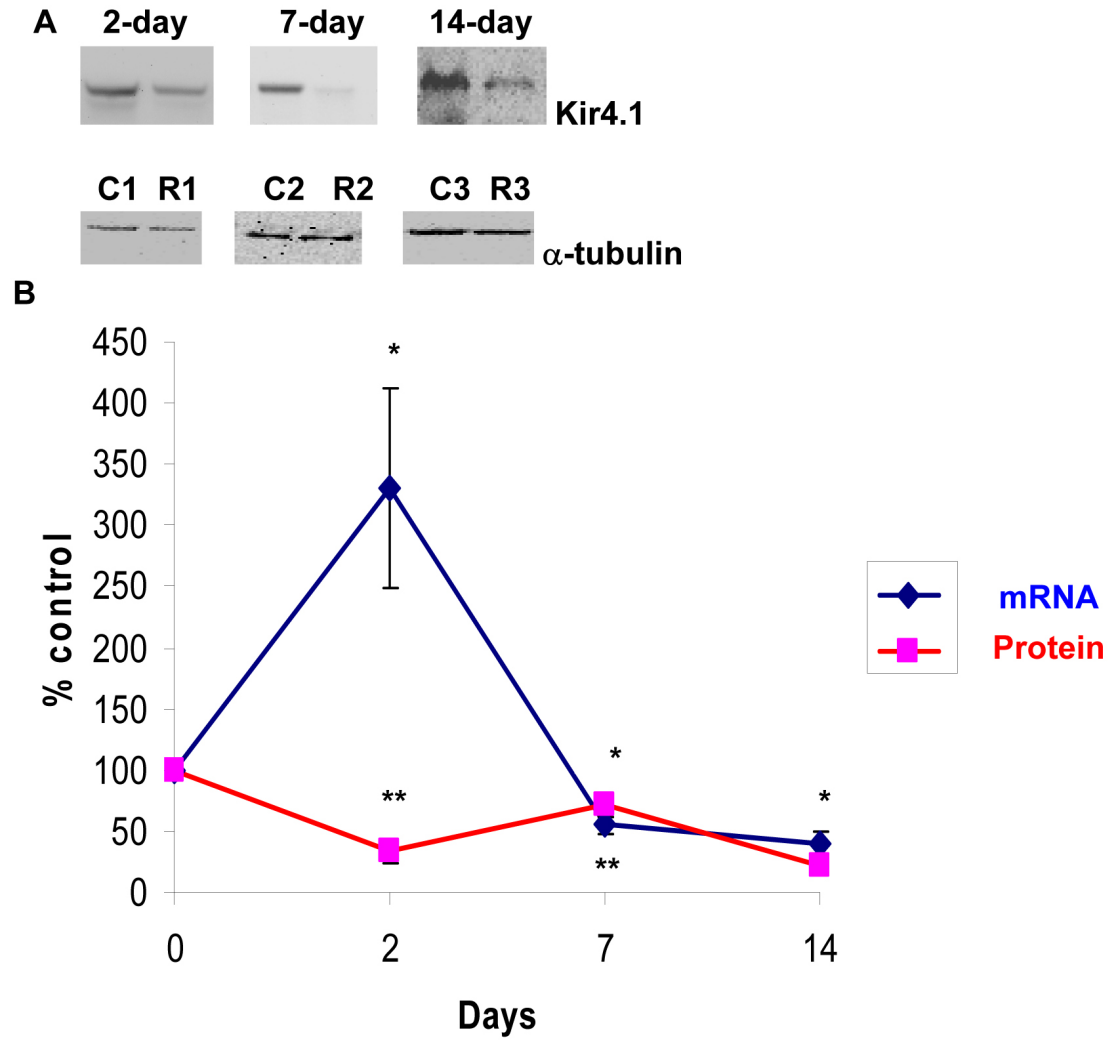
Optic nerve crush decreased Kir4.1 protein and mRNA levels. Following retinal injuries with optic nerve crush, retinas were dissected and plasma membrane proteins were isolated. Total RNA was isolated and transcribed into cDNA. Real-time PCR was performed using specific primers (see Methods). mRNA expression of *Kir4.1* was adjusted to the mRNA copies of *ACTB* (reference gene). Thirty microgram protein was loaded into each lane. Immunoreactive bands for Kir4.1 and β-tubulin 2, 7, and 14 days after optic nerve crush showing a significant reduction in Kir4.1 protein levels. Quantitative measurement using western blot showed that elevation of optic nerve crush decreased Kir4.1 protein levels (34±10, 72±6, 22±9, at 2, 7, and 14 day, respectively, **A**, n=7). Densitometric quantification is shown in **B**. Data are expressed as a ratio of the control value and each measurement represents mean±SEM *Denote statistical significance of Kir4.1 protein levels in optic nerve crushed retinas versus sham (p<0.005) as determined by one-way ANOVA and Tukey multiple comparison test. Results indicate that mRNA expression level of *Kir4.1* was significantly lower in optic nerve crushed retinas compared to sham at 7 and 14 days (55±7%, 41±9%, at 7, and 14 day, respectively, **B**, n=6). By contrast, optic nerve crush increased *Kir4.1* mRNA at 2 days (325±81%, **B**, n=6). **Denote significant differences compared with sham-retinas at p<0.05. Abbreviations: sham eye (C), and crushed (R).

In Müller cells, AQP4 co-localizes with an important retinal potassium channel known as Kir4.1 (mediates bidirectional K^+^ currents), and the co-expression of AQP4 with Kir4.1 suggests that water transport is coupled to the spatial buffering K^+^ currents [45,46]. The co-expression also suggests that osmotic gradients between the retina and the blood and vitreous can be compensated by K^+^ and water influxes and effluxes from Müller cells. Therefore, the effect of optic nerve crush on Kir4.1 expression in retina was tested. As shown in Figure 3A, ONC caused a significant reduction in Kir4.1 protein levels at all time points (34±10, 72±6, and 22±9, p=0.001 at days 2, 7, and 14, respectively, Figure 3A,B, n=7). However, while ONC resulted in an increase in *Kir4.1 *mRNA at day 2 (~300%), as determined by Q-PCR (325±81%, p<0.001 versus control, Figure 3B, n=6), it caused a significant decrease at days 7 and 14 (55±7% and 41±9%, respectively, p<0.001 versus control, Figure 3B, n=6). Changes in *Kir4.1* mRNA and protein levels are shown in Figure 3B.

### Effect of optic nerve crush on *AQP9* in rat retinas

**Figure 4:**
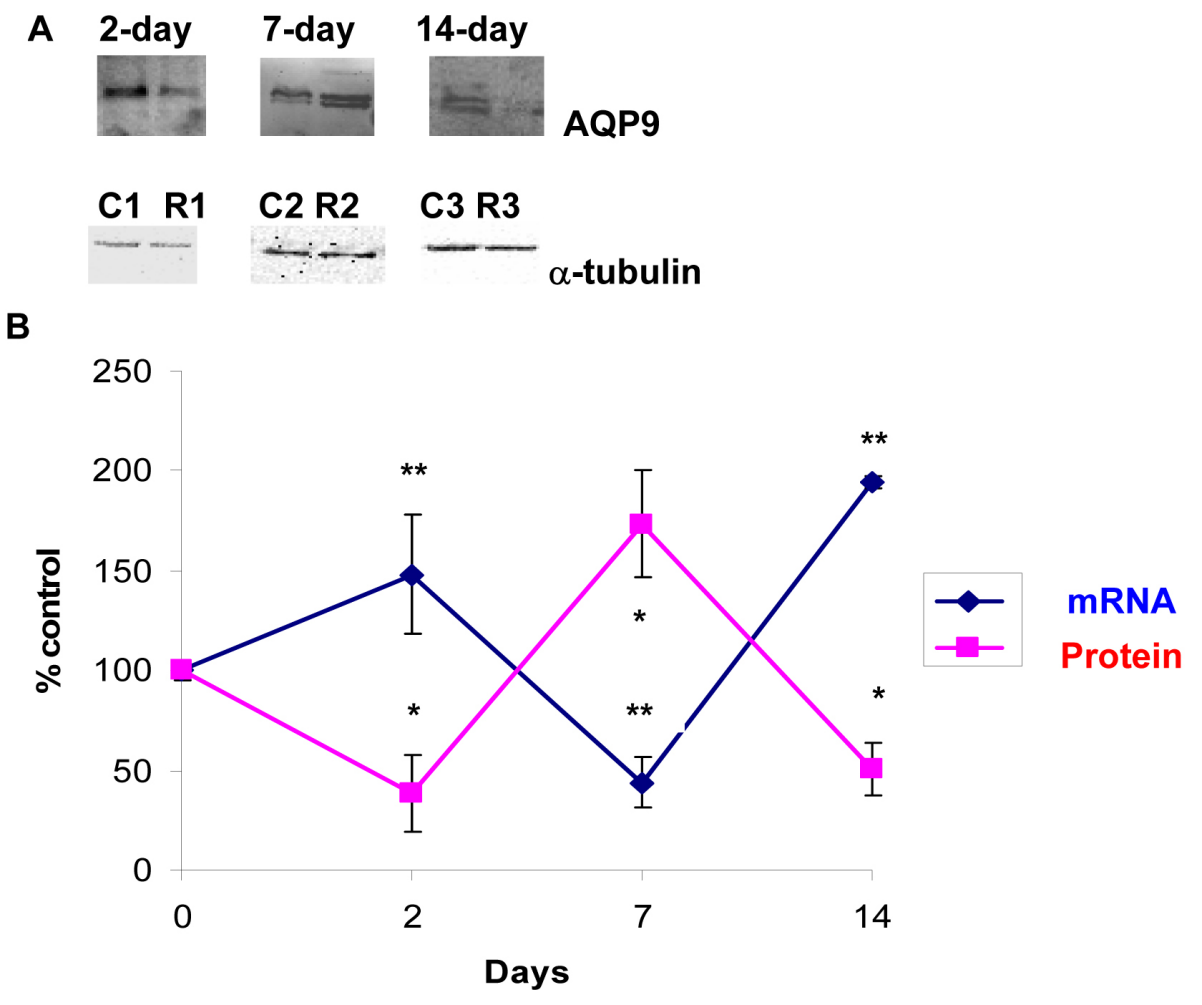
Optic nerve crush induced novel changes in AQP9 protein and mRNA levels. AQP9 protein levels showed opposite trend to changes in mRNA where they increased at 7 day (174±27%, p<0.001 versus control, **A**, n=6) but decreased at 2 and 14 day (38±19%, 50±13%, at 2, and 14 day, respectively, **A**, n=8). AQP9 shows a doublet and both bands were used in densitometric quantification in **B**. Data are expressed as a ratio of the control value and each measurement represents mean±SEM *Denote statistical significance of AQP9 protein levels in optic nerve crushed retinas versus sham (p<0.005) as determined by one-way ANOVA and Tukey multiple comparison test. Optic *AQP9* transcripts were quantified by quantitative real-time PCR (**B**). Gene expression data of *AQP9* is calculated after normalizing with *ACTB*. *AQP9* mRNA increased at 2 and 14 day (150±30%, 200±30%, at 2, and 14 day, respectively, n=5) but decreased at 7 day (44±13%, n=6, **B**). **Denote significant differences compared with sham-retinas at p<0.05. Abbreviations: sham eye (C), and crushed (R).

Several studies have shown that ONC induced hypoxia to the optic nerve [31-33]. A key channel that undergoes changes is AQP9, and its expression also appears to be upregulated after ischemic insult in the brain [47] and following IOP elevation (Dibas et al., unpublished observation). Therefore, the effect of ONC on AQP9 expression in retina was evaluated. Surprisingly, AQP9 protein levels showed an opposite trend to changes in message (mRNA) where they increased at day 7 (174±27%, p<0.001 versus sham, Figure 4A,B, n=6) but decreased at days 2 and 14 (38±19% and 50±13%, respectively, p<0.001 versus sham, Figure 4A,B, n=8). ONC resulted in novel changes in *AQP9*; its mRNA increased at days 2 and 14 (150±30% and 200±30%, respectively, p<0.001 versus control, Figure 4B, n=5) but decreased at day 7 (44±13%, p<0.001 versus sham, Figure 4B, n=6). The relationship between *AQP9* mRNA and protein changes is shown in Figure 4B.

### Effect of optic nerve crush on glial fibrillary acidic protein and bcl-xl in rat retinas

**Figure 5:**
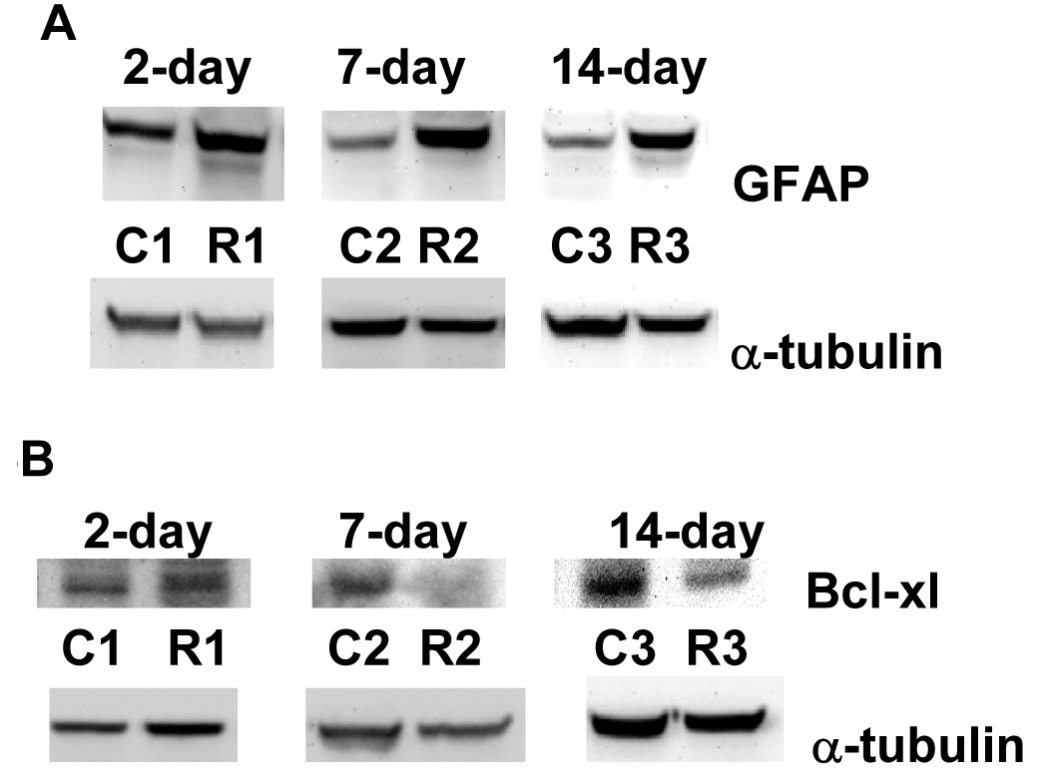
Glial fibrillary acidic protein is upregulated following optic nerve crush, while B cell lymphoma-x (bcl-xl) is downregulated. Following retinal injuries with optic nerve crush, retinas were dissected and cytosolic proteins were isolated. Fifty micrograms protein was loaded into each lane. Glial fibrillary acidic protein (GFAP), a cellular marker for retinal injury, was upregulated (**A**: 450 ±190%, 262±30%, and 300±34%, days 2, 7, and 14, respectively, p<0.001, n=7). Bcl-xl (anti-apoptotic factor) downregulation is involved in initiating apoptosis and not surprisingly was reduced following optic nerve crush (**B**: 67±9% and 56±10%, at days 7 and 14, respectively, p<0.005, n=7). However, changes in bcl-xl protein levels at day 2 were not statistically significant.

A universal early cellular marker for retinal injury is the upregulation of the intermediate filament protein GFAP, and its levels are commonly used as an index of gliosis [48]. As expected, ONC also resulted in an increase in GFAP protein levels that persisted at all time points (Figure 5A, 450±190%, 262±30%, and 300±34% at days 2, 7, and 14, respectively, p<0.005, n=7).

Finally, we tested the effect of ONC on bcl-xl (anti-apoptotic factor). Bcl-xl downregulation is involved in initiating apoptosis and not surprisingly was reduced following ONC (Figure 5B, 67±9% and 56±10% at days 7 and 14, respectively, p<0.005, n=7). However, changes in bcl-xl protein levels at day 2 were not statistically significant.

## Discussion

Human traumatic optic nerve injuries are usually the consequence of severe head trauma, with prospects for the recovery of vision being poor. Matsuzaki et al. [49] reported that when the visual acuity was zero at the time of the first examination, no visual improvement could be obtained. In an attempt to develop experimental models for traumatic optic neuropathy, several groups have used direct mechanical injury to the optic nerve to induce axonal damage and retinal ganglion cell loss. The ONC model closely mimics the damage that occurs in traumatic optic neuropathy, which results from an indirect trauma to the optic nerve due to an impact to the head. However, the crush model is regarded as an acute model of glaucoma [31-33].

Based on clinical and experimental studies, it appears that optic nerve damage involves multiple mechanisms that have yet to be elucidated [49]. Two key channels that affect retinal function are AQP4 (water) and Kir4.1 (potassium) channels. Both channels co-localize in distinct membrane domains of glial cells in retina [46] and brain [47]. However, AQP4 is expressed alone in the plexiform layer, whereas Kir4.1 is not. Both channels play important roles in ion homeostasis and “K^+^ spatial buffering” [50-57]. Retinal water transport or changes in extracellular space volume appear to parallel transglial K^+^ currents, and mice lacking AQP4 have a significant delayed cellular re-uptake of K^+^ from the extracellular space [46,58,59]. K^+^ spatial buffering within the inner retina occurs via the redistribution (siphoning) of excess K^+^ from the extraneuronal space toward fluid reservoirs of low K^+^ (or sinks), such as vitreous body, subretinal space, and blood vessels [60,61]. This is especially important since firing of neurons changes the extracellular concentration of K^+^ ions ([K^+^]o) due to excess K^+^ ions liberated from neurons into the intracellular space and causes a slow depolarization of glial cells, which have the ability to maintain [K^+^]o at a constant level. Spatial K^+^ buffering generates osmotic gradients, leading to the uptake of sodium and bicarbonate, which causes the intracellular osmolarity to increase and drives water into the glial cells through AQP4. An imbalanced reduction of AQP4 levels may therefore impair retinal K^+^ buffering, and uncontrolled increases in [K^+^]o may induce uncontrolled hyperexcitability and abnormal synchronization of retinal neurons. In the present study using the rat ONC model, we have shown for the first time that such injury causes a significant decrease in AQP4 and Kir4.1 protein and mRNA levels in retina, suggesting impaired ion homeostasis and K^+^ spatial buffering.

However, *AQP4* downregulation may be initially neuroprotective. *AQP4* deletion in mice is neuroprotective in a transient ischemia model of retinal injury [62], and the permanent middle cerebral artery occlusion model in rodents (similar to ischemic hemispheric stroke in humans) demonstrated that wild-type mice had a higher mortality rate and a significantly greater neurologic deficit at 24 h compared with AQP4 null mice [63].

Several studies have suggested that AQP4 levels vary depending on the insult being studied. For example, while middle cerebral artery occlusion and hyperosmotic stress induced by intraperitoneal infusion of mannitol increased AQP4 in rodent brain [64,65], hypoxia evoked a marked decrease in AQP4 in astrocytes in vitro, and subsequent re-oxygenation elicited the restoration of the expression of AQP4 to its basal levels [66].

Another key channel is AQP9. Although lactate can be transported by special proteins known as monocarboxylate transporters, it can also be transported by AQP9. AQP9 possesses general features of a water channel, but in addition it is permeable to a wide variety of noncharged solutes, such as lactate, β-hydroxybutyrate, glycerol, purines, pyrimidines, urea, mannitol, and sorbitol [67]. ONC exerted novel changes in *AQP9* mRNA and protein levels where it appears that increased mRNA slowed protein translation, and whenever mRNA decreased, protein production increased. Although the exact mechanism for such a relationship is unknown, it may reflect the retinal fuel supply and demand changes following a massive injury, such as crush, or an attempt at osmoregulation. It has been accepted that glucose conversion by Müller cells and astrocytes into lactate followed by its release into the extracellular space can serve as a fuel for neurons [68-72]. It is also thought that the glial–neuronal lactate shuttle is important for recovery of neurons after hypoxic injury as the blockade of lactate transport exacerbates neuronal damage in a rat model of cerebral ischemia [73]. While *AQP9* expression was upregulated after an ischemic insult [67], hypoxia evoked a significant decrease in AQP9 in astrocytes [66].

A universal early cellular marker for retinal injury is the upregulation of the intermediate filament protein GFAP. Although the exact function of GFAP is unknown, its immunoreactivity is commonly used as an index of gliosis [74]. The current study has shown an increase in GFAP protein levels (by western blot) following the ONC procedure. *GFAP* was upregulated following elevation of IOP [75-79], and increased GFAP staining was observed in astrocytes at the optic nerve head; this staining correlated strongly with the severity of glaucoma in POAG patients [74]. Furthermore, retinal injuries induced by intravitreal enthothelin injection increased GFAP expression in rat retina [42,80], which further confirms that GFAP is a universal indicator for cellular/tissue injuries and where glia replace dead neurones. When changes in thy-1 expression were compared to the time course of RGC cell loss, it is clear that a significant loss of *thy-1* mRNA and protein precedes RGC cell loss. Our results are in agreement with earlier reports showing that thy-1 is an early marker of RGC stress but not a marker of RGC loss and that *thy-1* mRNA and protein levels do not reflect the number of RGCs present in models of retinal damage [81-83].

In summary, in the current study we report changes in key AQPs (AQP4 and AQP9) and the K^+^ channel that may explain multiple mechanisms mediating optic nerve degeneration following mechanical injury that resembles human traumatic optic nerve injuries resulting from a severe head trauma. A better understanding of the cascade of events following optic nerve injuries will help provide better medical intervention and pioneer new therapeutics for prevention or restoring irreversible vision loss in patients after a traumatic optic nerve injury.
